# On the Optimization of Wireless Multimedia Sensor Networks: A Goal Programming Approach

**DOI:** 10.3390/s120912634

**Published:** 2012-09-17

**Authors:** Antonio-Javier Garcia-Sanchez, Felipe Garcia-Sanchez, David Rodenas-Herraiz, Joan Garcia-Haro

**Affiliations:** Department of Information and Communication Technologies, Universidad Politécnica de Cartagena, Campus Muralla del Mar, Cartagena E-30202, Spain; E-Mails: felipe.garcia@upct.es (F.G.-S.); david.rodenas@upct.es (D.R.-H.); joang.haro@upct.es (J.G.-H.)

**Keywords:** Wireless Multimedia Sensor Networks, optimization, network lifetime, throughput, goal programming

## Abstract

Network lifetime is a crucial concern for Wireless Multimedia Sensor Networks (WMSNs), particularly due to the energy constraints of their nodes and the significant bitrate required by multimedia applications. This paper deals with this issue, studying how to achieve the maximum network lifetime, and simultaneously satisfying the best aggregate throughput for the multimedia services offered. To this end, we propose a planning model that results in a more accurate solution for an arbitrary network deployment than using the current optimization techniques applied both to WMSNs and traditional Wireless Sensor Networks (WSNs). Our model is based on multi-objective formulation and goal programming, which, to the best of our knowledge, have never been employed in the WSN field. This paper also proposes a load balancing algorithm which ensures a fair traffic load distribution per link during the network operation and matches the values returned by the mathematical planning model for the set lifetime and throughput. Simulation results are presented and further discussed to show the effectiveness of the numerical solutions. Finally, a test-bed deployed in a trial environment validates the theoretical contributions of this work.

## 1. Introduction

A critical issue in energy-efficient Wireless Multimedia Sensor Networks (WMSNs) [1] design is how to maximize the throughput. This decision has a clear influence on the power required by the network nodes deployed in the area of interest. Unlike Wireless Sensor Networks (WSNs) focused on sensing monitoring of some physical parameters (temperature, pressure, humidity, *etc.*), WMSNs are intended to offer added-value services, e.g., tele-surveillance, which demand higher throughput and have a negative impact on the network lifetime in comparison with conventional WSN applications. On the one hand, maximizing the throughput objective enables these added-value services in the network, which also implies the improvement of technical aspects such as increasing the number of video sensors or the image quality. However, the network lifetime (defined as the time period between the network starts its operation and the first node depletes its battery) is jeopardized due to the notable increase of the nodes energy consumption. On the other hand, maximizing the network lifetime objective often involves worsening the performance of these added-value services.

Therefore, WMSN services exhibit conflicting objectives: when the throughput improves, the lifetime deteriorates, and *vice versa*. This explains why WMSNs have not been fully exploited yet, being currently an open issue of intensive research. In order to face this shortcoming, recent mathematical models based on optimization techniques were developed to balance both metrics. In this context, some works optimize both figures separately. They focus on either optimal bitrates entailing fixed network lifetime, or optimal lifetime assuming fixed transmission rates [2–4]. Other works [5,6] sort out this concern by optimizing throughput and lifetime sequentially, what may lead to a better performance of only one metric, but degrading the other one. These simplifications considerably relax the mathematical problem at the expense of deteriorating the results; therefore they do not obtain the optimal solution for the set of both parameters.

To solve these drawbacks and *as the main contribution of this work*, a network planning approach aimed at optimizing *both objectives simultaneously* is presented. This planning solution is applicable to any static WSN deployment (node mobility is not taken into consideration), but its major impact is on the WMSN field. It is based on the well-known multi-objective optimization (MO) method. The MO method allows one to formulate both objectives in the same problem definition, in order to later select one or several mathematical techniques which will solve the formulated problem. In particular, among all the possible mathematical tools, we select the Goal Programming (GP). As any other optimization technique, GP is used to introduce estimations about real network conditions (denoted as *goals*) into the constraints of the problem. However, the strong point of GP is its capacity for simultaneously optimizing the deviations (underachieve/overachieve) of these goals, expressing problems usually based on linear/non-linear MO programming. As a result, GP offers more flexibility to compute the optimal solution, in comparison with other recent WSN studies based on MO [7–10], which tackle similar problems but directly optimizing the metrics through a rigid and complex formulation. Thus, GP results in a more adjustable model to real network operation conditions, combining simplicity of formulation and feasibility to achieve the solution. This is the reason why GP is the selected multicriteria decision making tool used in a large quantity of scientific disciplines [11]. However, to the best of our knowledge, no previous work has dealt with GP in the WSN domain.

To assess our goal programming model, we estimated the goals for the throughput and network lifetime metrics which are consistent with the operation of some real scenarios related to agriculture and forest applications. The results obtained by GP guarantee an optimal design, including an efficient and complete sensing monitoring and tele-surveillance operation during, at least, the period demanded by the end-users for those WMSN applications.

In addition, this paper also contributes with the design of an *Optima****L*** *L****O****ad B****A****lancing Algorith****M***, denoted as LOAM, which is implemented and executed at every wireless network node. This algorithm determines, in runtime, the traffic load that must be transmitted to each network link as a function of the available battery level of the nodes. It obtains, as a result, throughput and network lifetime values similar to those calculated by the previous analytical planning model. To achieve this purpose, we take advantage of the collaboration among the network nodes and the exploitation of underused links, what leads to fairly balancing the network flows load (data and multimedia). Furthermore, this new algorithm is compatible with any static network topology employed (cluster, grid, random, *etc.*), demonstrating the flexibility and robustness of LOAM.

The numerical results have also been validated by means of computer simulations (executing the LOAM algorithm) and a real test-bed scenario implementation. Simulations confirm the feasibility of the proposed system and its behavior, giving the throughput and network lifetime results as a function of the traffic load applied. To this aim, a complete system simulator (including MPEG-4 video sources and CBR traffic for the remaining sensing nodes) has been programmed over the IEEE 802.15.4 standard [12] of the ns-2 framework [13]. Finally, thanks to the insight provided by the simulation results, a real video surveillance and sensing monitoring application has also been implemented and intensively tested. To do so, we have programmed hardware prototypes, based on the Imote2 wireless module [14,15] for video capture, and MicaZ devices [16] for sensing monitoring of physical parameters, which constitute the nodes of a WMSN deployed in an agriculture environment.

The rest of the paper is organized as follows: Section 2 summarizes the related work found in the open literature. In Section 3 the optimization problem is formulated and solved by means of the goal programming multi-objective technique, and the results obtained are analyzed. Our load balancing algorithm LOAM is presented in Section 4. Section 5 shows and comparatively discusses the performance evaluation results obtained from analysis and simulation. Section 6 describes the details of the real implementation and presents the experimental results measured, which further validate the former values. Finally, Section 7 concludes this paper.

## 2. Related Work

Due to the strict limitations of power supply, memory storage and processing capacity of the WSN devices, there is a large amount of scientific literature devoted to optimizing different metrics such as lifetime, latency or reliability [1,17]. However, most of these metrics are in conflict with each other, what leads to the need to solve complex problems. For this reason, most of the works reviewed simplify the problem formulation, just optimizing a single metric (e.g., [18,19]) or conducting a process where the selected metrics are optimized sequentially (not simultaneously). In order to do so, linear/non-linear programming techniques are used [2,3,6,20]. As a consequence, the solutions provided are not appropriate because the fully optimization of a metric does not imply optimal results for the other performance figures.

In this context, Hou *et al.* [2] presented a solution that fairly balances the rate allocation in hierarchical (cluster) topologies. To achieve it, the authors employ linear programming and polynomial-time algorithms to firstly maximize the information that each cluster-head can collect. This result is then introduced as a constraint into a second optimization step, aimed at maximizing the traffic load of all nodes until one or more nodes reach their energy-limited capacity for a given network lifetime requirement. In [3], a cross-layer architecture for WMSN is used to minimize the end-to-end delay and to maximize the total data gathered at the base station, both objectives are satisfied for a predefined network lifetime. To this aim, the authors firstly obtain a maximum transmission radius at the physical layer by using the minimum data generation bitrate. Secondly, this transmission radius is taken as a new constraint in a linear optimization problem to minimize the end-to-end delay. This outcome yields to the selection of the optimal routing paths. Finally, the maximum transmission radius is employed again to adjust the data generation bitrate of the source nodes in order to balance the data flow per link and maximize the data gathered at the base station. In both works [2,3], the lifetime is considered a constant value of the optimization problem. Therefore, lifetime is not an objective to be optimized, what restricts the scope of these works, which are thus very limited to specific applications where energy efficiency is not a concern [21]. In [6], the minimization of the energy consumption on each node, alongside with a throughput maximization problem, is solved sequentially by means of linear programming tools. Therefore, the best results are reached for the energy consumption but the maximum data gathered at the sink is not attained. Recently, Conesa-Muñoz *et al.* [20] solve a min-max problem in which firstly they maximize the sensing coverage, in order to later minimize the power consumption. The solution offered by this work shows a fixed scheme for data transmission which is not suitable for WMSN deployments.

To overcome the limitations of single or sequential-objectives optimization, some studies offer the best performance for a set of metrics. In this context, the multi-objective (MO) optimization is a well-known mathematical technique employed extensively in several scientific fields. This technique is based on the idea of optimizing a set of different conflicting metrics, which leads to optimal solutions satisfying all the objectives under consideration. To the best of our knowledge, most of the WSN works that employ MO techniques are focused on planning the optimal location of WSN nodes in the deployment of the network with the aiming of optimizing the network lifetime and network coverage without compromising the connectivity among nodes. An in depth study of many of these works may be found in [22]. More recent investigations deal with this issue, such as [7] and [23], which introduce the new guidelines for MO techniques to obtain an optimal deployment as well as maximize the network lifetime. Martins *et al.* [23] propose the Multi-objective On line Hybrid Algorithm (MultiONHA), a combination of: (1) a genetic algorithm [24] denoted as Multiobjective Global on-Demand Algorithm (MGoDA) which, in turn, is based on the non-dominated Sorting Genetic Algorithm 2 (NSGA-II) [25]; and (2) a deterministic approach, named Local on line Algorithm (LoA). Both schemes, MGoDA and LoA, are exclusively implemented at the base station. MGoDA is employed to handle the active/inactive periods (duty cycle) [26] of the nodes in order to build different routing paths in a tree fashion. This routing algorithm guarantees an optimal network lifetime and satisfies the connectivity between any source node and the base station. On the other hand, LoA uses neighboring information to perform network recovery tasks when an unpredictable failure occurs (e.g., nodes depleting their batteries). However, the time and energy consumption associated with these recovery tasks represents a serious drawback for its real implementation in the WSN devices.

Following the line of recent works about optimal WSN deployment, a hybrid multi-objective framework is presented in [7]. Konstantinidis *et al.* proposed the Multi-Objective Evolutionary Algorithm based on Decomposition (MOEA/D) [27], which decomposes a multi-objective problem into a collection of sub-problems resulting in a set of Pareto-optimality [28]. From this set of solutions (which comprises a Pareto front), the authors follow six different single-objective strategies to select the best solution according to the requirements imposed by the application. These requirements are divided into three categories: (1) all objectives are simultaneously optimized; (2) the solution that offers the highest value of network lifetime is selected; and, finally, (3) the solution chosen offers the greatest network coverage. A strength of the proposal [7] is the balancing of the network traffic load to avoid jeopardizing the battery level of those nodes that dispatch a large amount of information. However, load balancing is not maximized in any case, since it is not an optimization objective. This involves the inability of optimizing the data flows/rates in each routing path and therefore data gathering at the base station is not optimal. Furthermore, the main focus of this work is on demonstrating that the algorithm's performance (in terms of its efficiency and convergence speed) is better than that offered by NSGA-II. As a result, although the authors proposed a case study for a WSN deployment, it lacks a complete performance evaluation of the network metrics of interest, such as throughput or lifetime.

In contrast to the large number of works about the optimal deployment of nodes, a few MO investigations deal with the optimization of other metrics as throughput and latency, as well as energy consumption. He *et al.* [8] presented a general mathematical framework for WSN, in order to address different types of multi-objective optimization problems in a distributed fashion. That is, each network node implements this framework and solves the MO problem independently, giving as a result a global optimal solution. A general MO problem is formulated by means of the Lagrange dual decomposition and stochastic programming [29]. Then, this problem is solved by using different MO optimization methods. In order to demonstrate the feasibility of the proposed framework, the authors solved, as an example, a MO problem which consists of maximizing the throughput at the sink and the network lifetime. To this end, three MO optimization methods are employed with different goals: (1) the *constraint method*, which only maximizes the throughput, becoming the network lifetime objective an additional constraint of the problem (this same procedure is applied in [30]); (2) the *linear weighted method* [31] maximizing both objectives simultaneously; and (3) the *hierarchical sequence method* [9] which first maximizes the throughput and then the network lifetime. Simulations are also conducted for a few nodes scenario (in particular, nine nodes), all of them in coverage. The main drawback of this model is its generalization to any figure of merit by means of the Lagrange decomposition, which guarantees the optimization convergence (since the generic functions derived are convex), but does not provide good precision for the figures under study. Therefore, the computed solution does not ensure achieving the optimal values for the referred figures. This fact is reflected in the poor results obtained in their simulations. Studies as [32] and [33] present multi-objective solutions for the optimization of the message delivery latency and energy consumption WSN metrics. The work in [32] describes a complex topology based on multi-tiers, where a set of best solutions (Pareto front) capturing the trade-off between both metrics is computed. However, it does not offer any tool for its validation. The research in [33] uses min-max techniques to solve the same optimization problem. In this case, the validation is carried out by means of the development of a simulation framework over the IEEE 802.11 standard, not taking advantage of the de facto standard for WSN, the IEEE 802.15.4. In contrast to our work, focused on optimizing two conflicting metrics, References [32] and [33] deal with two compatible metrics since the minimization of the message latency entails a reduction of the ON/active periods of the duty cycle at the nodes, and, as a result, the improvement of the network lifetime.

On the other hand, concentrating on the WMSNs field, research papers [9,10,34] employ MO optimization. In particular, the work described in [10] proposes a distributed algorithm to control multiple objectives, such as latency, bandwidth or network lifetime, among others. For this purpose, the authors used the Ant Colony Optimization (ACO) theory [35], which supplies a set of possible solutions for this MO problem thanks to the transmission of control messages denoted as *ants*. They test all possible routing paths towards the base station. Then, a genetic algorithm running in each node optimizes these paths and selects those in accordance with the optimization results. The main weakness of this work is the excessive overhead and energy consumption necessary for the transmission of *ants* which flood the network. Furthermore, due to the limited memory and processing capabilities of the network nodes, a high delay is incurred in order to find the optimal paths, thus deteriorating the optimal solution for the objectives under study.

Finally, EkbataniFard *et al.* [34] employed the NSGA-II algorithm to optimize the end-to-end delay, reliability, and network lifetime objectives. The algorithm is only implemented at the base station, which is informed about the placement of the devices and all possible routing paths from any source node. This information is used by NSGA-II to calculate a set of Pareto-optimal solutions to later select the best one depending on the application requirements. Lai *et al.* [9] defined a non-linear multi-objective optimization problem to solve the concern addressed in [2], but considering the network lifetime as an objective to optimize. To this end, the authors follow a lexicographical order [36], where the maximization of lifetime is prioritized over the maximization of the data gathered at cluster-heads, thus solving the flow balancing problem described in [2]. Both approaches [9] and [34] are applied to cluster-tree networks, because this topology greatly facilitates the development of multimedia applications where streaming contents flow from different source nodes to their respective cluster-head in one-hop. Then, multimedia data is dispatched to the next cluster-head closer to the base station. Thereby, only with the appropriate coordination of different cluster-head nodes, the multimedia flow is routed from border nodes to the base station. The main weakness of this work is inherent to the exploitation of the cluster-tree topologies since they unbalance the traffic load among all network nodes. In this sense, the authors point out that the network lifetime is only influenced by the energy consumption of the cluster-heads because they handle all network traffic, thus consuming more energy than any another node (bottleneck nodes).

Our research introduces a multi-objective programming technique in topologies where nodes within the same hierarchy may communicate with any neighbor in coverage, sharply reducing the number of bottleneck nodes of the network. Furthermore, as far as authors know, it is the first time that goal programming is introduced in the WSNs/WMSNs field to solve the multi-objective problem. This technique incorporates the concept of flexibility into the objective functions which leads to more accurate solutions, as far as realistic network conditions are concerned, than the ones offered by the works also summarized in this section.

## 3. Problem Formulation: Multi-Objective Optimization through Goal Programming

Throughput and lifetime are two conflicting metrics because an increase in the achieved throughput in the network links causes more energy consumption per node, worsening the network lifetime. In contrast, a decrease in power consumption of the nodes can be accomplished by reducing, among other metrics, the achieved throughput per link. Therefore, *a priori*, the optimization of both metrics simultaneously is unfeasible.

Reviewing the specialized literature, the multi-objective optimization concept described in [37] shows a powerful mathematical method where a set of *objective functions* are evaluated normally by specifying linear/non-linear multi-objective programming. This method applied to our problem would satisfy, a priori, the optimal value for the set throughput and lifetime, achieving in turn their best performance for any network topology.

In our model, two metrics are maximized: (i) the network lifetime defined as the minimum node lifetime for any node different to the sink/base station and (ii) the aggregate throughput characterized as the total bitrate (sum) of all network links. For this purpose, the multi-objective programming is applied taking into account the following assumptions:

- Random topologies are selected (but nodes are fixed in its position).
- The information flows from any network node to a unique sink or base station (in our case, a special node without energy limitations).
- Any node may transmit data to its neighbors in coverage closer to the sink.
- Two types of WMSN nodes are considered: video and sensing nodes.
- The optimization process finalizes when one or more nodes reach their energy-constrained capacities.

Under these assumptions, the MO formulation for our model is as follows:

(1) $$\text{Maximize}\;\Lambda =\sum_{i=2}^{N}\sum_{j=1}^{N}{\text{th}}_{ij}^{V}\cdot K_{ij}\cdot S_{i\to j}+\sum_{i=2}^{N}\sum_{j=1}^{N}{\text{th}}_{ij}^{D}\cdot K_{ij}\cdot S_{i\to j}$$

(2) $$\begin{array}{cc} \text{Maximize} & T_{\text{NETWORK}} \end{array}$$

subject to:

(3) $${\text{th}}_{ij}^{V}>0,{\text{th}}_{ij}^{D}>0,T_{\text{NETWORK}}>0,\forall (i,j)\in N,i\neq j$$

(4) $$\begin{array}{l} \sum_{j=1}^{N}{\text{th}}_{ij}^{V}\cdot K_{ij}\cdot S_{i\to j}+\sum_{j=1}^{N}{\text{th}}_{ji}^{V}\cdot K_{ji}\cdot S_{j\to i}+\sum_{j=1}^{N}{\text{th}}_{ij}^{D}\cdot K_{ij}\cdot S_{i\to j}+\sum_{j=1}^{N}{\text{th}}_{ji}^{D}\cdot K_{ji}\cdot S_{j\to i}\leq \nu \\ \forall i\in N,i\neq 1,i\neq j \end{array}$$

(5) $$\begin{array}{l} E_{tx}\cdot \sum_{j=1}^{N}{\text{th}}_{ij}^{V}\cdot K_{ij}\cdot S_{i\to j}+E_{rx}\cdot \sum_{j=1}^{N}{\text{th}}_{ji}^{V}\cdot K_{ji}\cdot S_{j\to i}+E_{tx}\cdot \sum_{j=1}^{N}{\text{th}}_{ij}^{D}\cdot K_{ij}\cdot S_{i\to j}+\\ E_{rx}\cdot \sum_{j=1}^{N}{\text{th}}_{ji}^{D}\cdot K_{ji}\cdot S_{j\to i}+E_{\text{Re}\;\text{sidual}}\leq \frac{E_{\text{source}}}{T_{\text{NETWORK}}};\forall i\in N,i\neq 1,i\neq j,E_{\text{source}}>0 \end{array}$$

(6) $${\text{th}}_{ij}={\text{th}}_{ij}^{V}+{\text{th}}_{ij}^{D}$$

(7) $${\text{th}}_{ij}\leq \Psi$$

where the different parameters are described in Table 1.

Table 1 shows the parameters employed in the multi-objective problem formulation. Expressions (1) to (7) are the multiple *objective functions* and *inequality/equality constraints* for the network lifetime and aggregate throughput joint maximization problem:

- Expression (3) avoids any throughput and lifetime lower than zero.
- Expression (4) represents the throughput conservation for each node, *i.e.*, the summation of the outgoing throughput plus the incoming throughput of an arbitrary node *i* must be less than or equal to the maximum transmission data rate offered by this node.
- Expression (5) models the energy constraints of a node which operates under an ON-OFF regime.
- Expression (6) points out to the total throughput conservation per link.
- Expression (7) limits the achieved throughput per link to the maximum transmission rate specified by the IEEE 802.15.4 standard.

To sum up Equations (1)–(7) provide, as a result, the achieved throughput per link, maximizing the aggregate throughput of the network and its lifetime, which can be interpreted as a useful planning outcome for the user/decision-maker. This means that, for each topology designed by the user, *a new solution for the traffic load per link is generated*. However, as the problem is currently formulated, it is not feasible to solve both metrics simultaneously. Therefore, in the next subsection, a mathematical tool, based on GP is proposed to solve these previous concerns.

### 3.1. Goal Programming Formulation

In many real applications, users/decision makers may estimate, *a priori*, the value for the goals to achieve. In our study, these values are established as lower bounds. For instance, in a WMSN deployment for a farming crop, we can estimate that the lifetime of WMSNs should comprise, at least, the entire process of cultivation and harvesting of the product under study. In other scenarios, for instance in a forest environment, the duration of WMSN must usually be longer because often the area has a difficult access, making a frequent substitution of the nodes' battery unfeasible.

The inclusion of lower and upper bounds guarantees the convergence of the problem defined by Equations (1)–(7). To confirm this proposition, the energy consumption and throughput per link metrics have been ranged from a minimum to a maximum value. In particular, the energy consumption is limited by the initial energy *E_source_* as upper bound and by the minimum level of battery power of each node when the network finalizes its operation. This latter value is calculated as the minimum level of energy required by a device/node to reach the expected lifetime (estimated by the decision maker). Within this range, any energy consumption expression is continuous and convex. According to this general guideline, the particular expression of Equation (5) must present an optimal value within the range under study. On the other hand, the throughput per link has the restriction imposed by the traffic generated (*v*) as lower limit, and the nominal bandwidth of the network (Ψ) as upper bound. These bounds must be fulfilled regardless of the type of traffic generated (sensing/monitoring data or video traffic). Therefore, once the value of the throughput per link is bounded, the expression (4) must contain a solution too. Finally, the aggregate throughput at expression (1) is upper bounded by the sum of the nominal bandwidths of all available links and it is lower bounded by zero, which is the lack of data transmissions. This fact assures the convergence of our calculations because the outcomes must be comprised between these bounds.

Analyzing the scientific literature, reference [8] is the main contribution which tries to offer a solution, optimizing the set of objectives aggregate throughput and lifetime for any network layout. However, the results obtained in [8] are poor as it was discussed above. Under these circumstances, **Goal Programming** (GP) is an appropriate multi-objective optimization technique focused on finding a solution that simultaneously satisfies both objectives. GP fulfills the target of finding a practical, real-world solution to a problem, transforming the objectives into goals. To this end, the decision makers preliminary estimate the expected result for that goal.

The idea behind distinguishing GP from conventional methods of optimization (*i.e.*, single-objective, sequential-objectives or other MO techniques) is to introduce flexibility into the objective functions (as opposed to the rigid constraints of the conventional techniques). Most of the traditional approaches to real-world decision problems uses models that usually presume the optimization of objectives subject to a set of rigid constraints [2,3,6]. This fact may suppose a deviation in the results related to the expected ones. GP entails an attempt to eliminate or, at least, mitigate this concern.

Mathematically, the goal programming problems are formulated as follows: (i) given *n objective functions Z(x)* = *Z_1_(x), Z_2_(x), Z_3_(x)*,…, *Z_n_(x)* where *x* ∈ *R^m^* define the decision variable vector and (ii) given the goal vector *z_goal_* = *[z_1,goal_ z_2,goal_ z_3,goal_*,…..,*z_n,goal_]* where *z_q,goal_* defines the goal for the objective *q*, the solution offered by the method is the *feasible point* nearest to the *z_q,goal_*. Thereby, the analytical model representing the optimization problem is:

(8) $$\begin{array}{l} \min\sum_{q=1}^{N}\left|z_{q,\text{goal}}-Z_{q}(x)\right|\\ \text{subject to}:x\in X\subseteq R^{m} \end{array}$$

where *X* and *x* denote the *feasible region* and the *feasible point* of the optimization problem, respectively.

The *objective function* expressed by Equation (8) is not linear and not differentiable. However, expression (8) can be converted into a linear function by introducing two positive deviational variables which represent overachieving or underachieving of the desired level for each goal [11]:

- $d_{q}^{+}$ denotes the overachieving level of the goal q.
- $d_{q}^{-}$ indicates the underachieving level of the goal q.

Furthermore, the method has to satisfy the condition $d_{q}^{+}\times d_{q}^{-}=0$. This means that $d_{q}^{+}$ and $d_{q}^{-}$ do not belong to the *basic solution* simultaneously because the *feasible point* cannot exceed or be lower than a goal. On the other hand:

(9) $$\left|d_{q}^{+}\right|+\left|d_{q}^{-}\right|=\left|z_{q,\text{goal}}-Z_{q}(x)\right|,\text{q}=1,\ldots \ldots ,\text{n}$$

(10) $$\left|d_{q}^{+}\right|-\left|d_{q}^{-}\right|=\left|Z_{q}(x)-z_{q,\text{goal}}\right|,\text{q}=1,\ldots \ldots ,\text{n}$$

(11) $$d_{q}^{+},d_{q}^{-}\geq 0,\text{q}=1,\ldots \ldots ,\text{n}$$

Using Equations (9), (10) and (11), the optimization problem expressed by Equation (8) is rewritten as follows:

$$\min\sum_{q=1}^{N}d_{q}^{+}+d_{q}^{-}$$

subject to:

(12) $$\begin{array}{c} Z_{q}(x)+d_{q}^{-}-d_{q}^{+}=z_{q,\text{goal}},\text{q}=1,\ldots \ldots ,\text{n}\\ x\in X\subseteq R^{m},d_{q}^{+},d_{q}^{-}\geq 0,\text{q}=1,\ldots \ldots ,\text{n} \end{array}$$

From Equation (12), our optimization problem is transformed to simultaneously maximize the aggregate throughput and the network lifetime. Thus, the problems (1)–(7) are re-formulated accordingly as follows:

$$\text{Maximize}\sum_{i=2}^{N}\sum_{j=1}^{N}d_{ij}^{+}+d_{N}^{+}$$

subject to:

(13) $$\begin{array}{l} \sum_{i=2}^{N}\sum_{j=1}^{N}({\text{th}}_{ij}^{V}+d_{ij}^{-}-d_{ij}^{+})\cdot K_{ij}\cdot S_{i\to j}+\sum_{i=2}^{N}\sum_{j=1}^{N}({\text{th}}_{ij}^{D}+d_{ij}^{-}-d_{ij}^{+})\cdot K_{ij}\cdot S_{i\to j}=z_{th,\text{goal}}\\ \begin{array}{cc} & T_{\text{NETWORK}}+d_{N}^{-}-d_{N}^{+}=z_{\text{Lifetime},\text{goal}} \end{array}\\ \begin{array}{cc} & \sum_{i=2}^{N}\sum_{j=1}^{N}d_{ij}^{+}\geq 0,\sum_{i=2}^{N}\sum_{j=1}^{N}d_{ij}^{-}\geq 0,d_{N}^{+}\geq 0, \end{array}d_{N}^{-}\geq 0,{{\text{th}}^{V}}_{ij}>0,{{\text{th}}^{D}}_{ij}>0,T_{\text{NETWORK}}>0,\forall (i,j)\in N,i\neq j\\ \begin{array}{ll} & \sum_{j=1}^{N}{\text{th}}_{ij}^{V}\cdot K_{ij}\cdot S_{i\to j}+\sum_{j=1}^{N}{\text{th}}_{ji}^{V}\cdot K_{ji}\cdot S_{j\to i}+\sum_{j=1}^{N}{\text{th}}_{ij}^{D}\cdot K_{ij}\cdot S_{i\to j}+\sum_{j=1}^{N}{\text{th}}_{ji}^{D}\cdot K_{ji}\cdot S_{j\to i}\leq \nu \\ & \forall i\in N,i\neq 1,i\neq j \end{array}\\ \begin{array}{ll} & E_{tx}\cdot \sum_{j=1}^{N}{\text{th}}_{ij}^{V}\cdot K_{ij}\cdot S_{i\to j}+E_{rx}\cdot \sum_{j=1}^{N}{\text{th}}_{ji}^{V}\cdot K_{ji}\cdot S_{j\to i}+E_{tx}\cdot \sum_{j=1}^{N}{\text{th}}_{ij}^{D}\cdot K_{ij}\cdot S_{i\to j}+\\ & E_{rx}\cdot \sum_{j=1}^{N}{\text{th}}_{ji}^{D}\cdot K_{ji}\cdot S_{j\to i}+E_{\text{Re}\;\text{sidual}}\leq \frac{E_{\text{source}}}{T_{\text{NETWORK}}}\forall i\in N,i\neq 1,i\neq j,E_{\text{source}}>0 \end{array}\\ \begin{array}{cc} & {\text{th}}_{ij}={\text{th}}_{ij}^{V}+{\text{th}}_{ij}^{D} \end{array}\\ \begin{array}{cc} & {\text{th}}_{ij}\leq \Psi . \end{array} \end{array}$$

where:

*z_th,goal_* is the goal for the aggregate throughput
*z_Lifetime,goal_* is the goal for the network lifetime
$\overset{N}{\underset{i=2}{\text{∑}}}\overset{N}{\underset{j=1}{\text{∑}}}d_{ij}^{+}$ denotes the aggregate throughput in excess of the goal *z_th,goal_*
$\overset{N}{\underset{i=2}{\text{∑}}}\overset{N}{\underset{j=1}{\text{∑}}}d_{ij}^{-}$ specifies the aggregate throughput short of the goal *z_th,goal_*
$d_{N}^{+}$ is the excess/positive deviation variable for the goal *z_Lifetime,goal_*
$d_{N}^{-}$ is the negative deviation for the goal *z_Lifetime,goal_*

Solving problem (13), the multi-objective formulation expressed by Equations (1)–(7) is also solved. Problem (13) provides an accurate linear multi-objective representation of the original formulation thanks to the estimation of the goal levels for the metrics under consideration. As a result, maximizing $\overset{N}{\underset{i=2}{\text{∑}}}\overset{N}{\underset{j=1}{\text{∑}}}d_{ij}^{+}+d_{N}^{+}$ satisfies the aggregate throughput and the expected network lifetime. In case these results were unfeasible, that is, the throughput, the lifetime or both were lower than the goals defined, our model decides on maximizing the underachieving levels of the goals (as it is shown by Equations (14) and (15)), maintaining the constraints of the problem (13). The objective is to search for the results $\overset{N}{\underset{i=2}{\text{∑}}}\overset{N}{\underset{j=1}{\text{∑}}}d_{ij}^{-}$ or $d_{N}^{-}$ that better adjust to the expected goals. In many occasions, this solution is valid because it is in accordance with the requirements of the real-problem proposed. Under these same circumstances, that is, the results reached were unfeasible, the conventional optimization techniques employed in WSNs/WMSNs [8,9,23,34] solve this problem by defining a new mathematical model where the design parameters must be reconsidered (for instance, removing video devices or modifying the placement of nodes). As it is shown, in comparison with the conventional techniques, GP may offer a solution without modifying the design parameters thanks to a higher flexibility in the analysis which is due to the use of deviation variables:

(14) $$\text{Maximize}\sum_{i=2}^{N}\sum_{j=1}^{N}d_{ij}^{-}+d_{N}^{+}\;\text{or Maximize}\sum_{i=2}^{N}\sum_{j=1}^{N}d_{ij}^{+}+d_{N}^{-}$$

(15) $$\text{Maximize}\sum_{i=2}^{N}\sum_{j=1}^{N}d_{ij}^{-}+d_{N}^{-}$$

Finally, for any new scenario under study, the solution of Equation (13) returns the traffic load of all network links. In detail, we can observe that the solution given by solving problem (13) fairly balances the achieved throughput per link, thus maximizing the data gathered at the base station. Therefore, our solution can be used as a planning tool and applied to any arbitrary topology (cluster-tree, grid, random, *etc.*).

### 3.2. Numerical Results

We start by estimating the *z_th,goal_* and *z_Lifetime,goal_* goal values. First, to calculate *z_th,goal_*, we have to consider the type of traffic and the average number of hops to the sink for any arbitrary pair source-destination (sink) nodes. Concerning the former, two types of traffic generation sources (*v*) have been included: (i) video nodes transmitting MPEG-4 video streaming at rates of 60 kbps and intervals of 1 min/hour, and (ii) sensing nodes dispatching sensor monitoring data at rates of 1 message per second. Video sources are strategically located to cover the entire observing area. On the other hand, and as can be seen in the work of Shu *et al.* [38], an intermediate node sharing several video flows entails that the video streaming quality perceived by the end-users is, in general, poor. To overcome this drawback, in our work, each video source generates video streaming at non-overlapping periods with the remaining video nodes. Our proposed scheme assumes that the video traffic generation pattern is known, selecting the same one that was satisfactorily tested in [39]. Each image is divided into messages of 127 bytes long (114 bytes of payload), the maximum size supported by the IEEE 802.15.4 standard. According to our previous experiences [39], the video bitrates, the payload and the video generation pattern selected are enough to periodically check the focus zone with high quality, provisioning a video streaming of 2–3 images per second with an image size similar to a mobile display (176 × 144 pixels and 1 byte per pixel). For the sensing nodes, 5 bytes of payload are used to monitor any physical parameter (temperature, humidity, pressure, *etc.*). As regards the number of hops to the sink, we have taken into account the place of each network node into the topology to calculate the average number of hops to reach the sink. Finally, no link must exceed the transmission data bitrate of 250 kbps (Ψ) defined by the standard.

As for *z_Lifetime,goal_*, we estimate, a priori, three possible values: 6, 9 and 36 months. The first one is a value given by agriculture experts/decision makers and it covers a high variety of farming (melon, *Cucumis melo* L., broccoli, *Brassica oleracea*, *var. italic*, tomato, *Solanaceae Lycopersicum Esculentum*, *etc.*) located in the Campo de Cartagena (Southeast of Spain) and the surrounding area. The second value taken into consideration corresponds to the maximum interval of time for replacing the batteries of the sensor devices in a forest area (data collected by the *Council of Environment and Territory Management of the Murcia Region*). Finally, the last value characterizes the deployment of small size networks operating during a noticeable long life on the focus zones under study.

**Figure 1.**
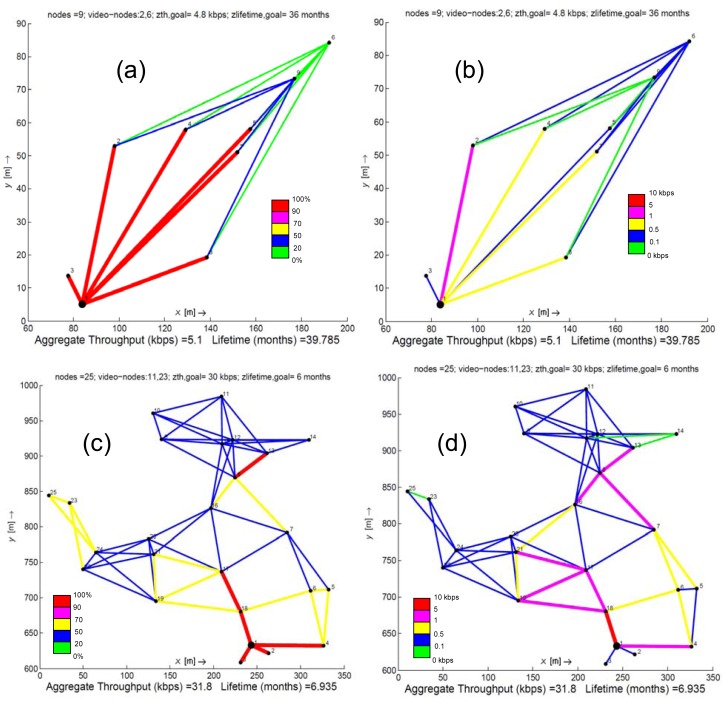
Results obtained for two random topologies consisting of nine and 25 nodes, respectively. Figures 1(**a**) and 1(**c**) present the % traffic load distributed by each node. Figures 1(**b**) and 1(**d**) plot the achieved throughput per link (kbps).

**Figure 2.**
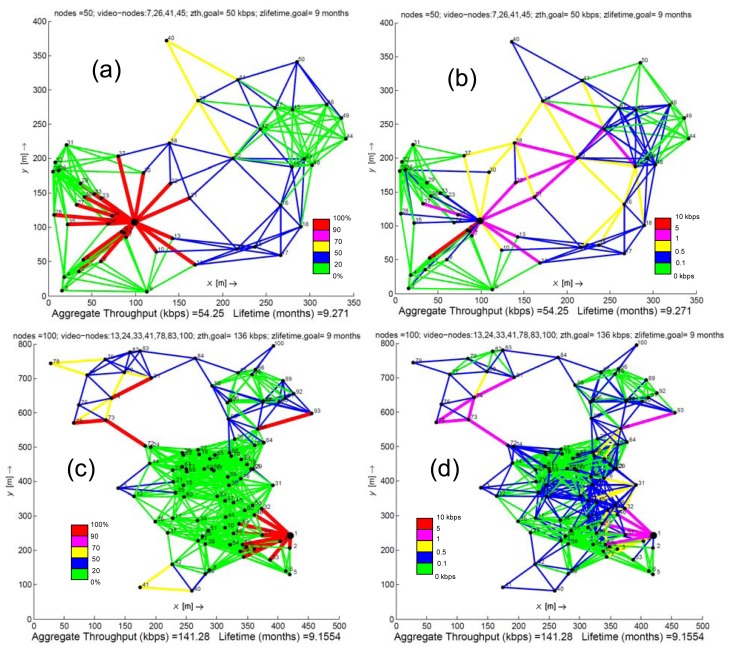
Results obtained for two random topologies of 50 and 100 nodes, respectively. Figures 2(**a**) and 2(**c**) present the % traffic load distributed by each node. Figures 2(**b**) and 2(**d**) plot the achieved throughput per link (kbps).

Figures 1 and 2 show the results given by solving problem (13). Both figures refer to topologies consisting of a fixed number of nodes. In particular, Figure 1 presents two scenarios which have been selected by its relevance in monitoring and observing all the usual agriculture processes of traditional crops located in the Campo de Cartagena. On the contrary, due to the big extensions of land to be monitored, the scenarios illustrated in Figure 2 are thought as appropriate for a forest area. The 50 nodes scenario is an ideal model for zones with a limited number of obstacles (trees, bushes/scrub, rocks, *etc.*) while the scenario of 100 nodes is focused on wooded areas.

Figures 1(a), 1(c), 2(a), and 2(c) depict the resulting traffic load for each network node. This can be interpreted as a traffic planning outcome by solving problem (13), where each network node out of the coverage of the sink fairly dispatches its messages to its neighbors (a link between nodes exists if both nodes are in coverage and data progress along the path to the sink), achieving load balancing. In these figures we observe that, for the case of crops or forest areas with scarce vegetation, a minimum number of nodes are enough to monitor these zones, but at the expense of dispatching more traffic load than nodes with more neighbors in their vicinity. This fact is clear in the topologies of nine, 25 and 50 nodes, where subzones formed by a few nodes concentrate a higher amount of messages from other subzones of the network. In these cases, the solution of problem (13) guarantees a fair balance of the load when there are redundant paths to the sink. For instance, in the scenario of 50 nodes, we can observe that the node denoted as *20 balances* its load to the nodes labeled as *21*, *22* and *38*, increasing their lifetimes and therefore, the network lifetime as well. In contrast, the scenario formed by 100 nodes shows a high concentration of nodes in a small area. In this context, solving problem (13) also achieves the best balancing of the traffic load among all the network links, what avoids overloading a few nodes that would increase their energy consumption.

Observing Figures 1(b), 1(d), 2(b) and 2(d), we can remark on three main aspects: (i) the high number of messages generated by the video sources are assimilated/dispersed by the nodes to decrease the impact of video streaming on the network lifetime (ii) the number of messages increases according to the proximity to the sink and (iii) as it was described in the previous paragraph, nodes with few neighbors usually retransmit more messages than nodes with more neighbors in their vicinity. Under these three premises, we conclude that links employed by video sources, by neighbor nodes of the sink or by nodes with few neighbors, collect more throughput than the remaining links. This means that nodes associated to these more active links are the ones with a better chance to deplete their batteries earlier.

Figures 1 and 2 also show that the aggregate throughput is slightly greater than the one established by *z_th,goal_*. This means that the average number of hops calculated from the solution of problem (13) is the same (or rarely one more hop) than the one estimated from calculating the *z_th,goal_*. Therefore, a large amount of messages generated by a same source node keeps the same number of hops as the one established for *z_th,goal_*, though they go through different paths to reach the sink.

**Figure 3.**
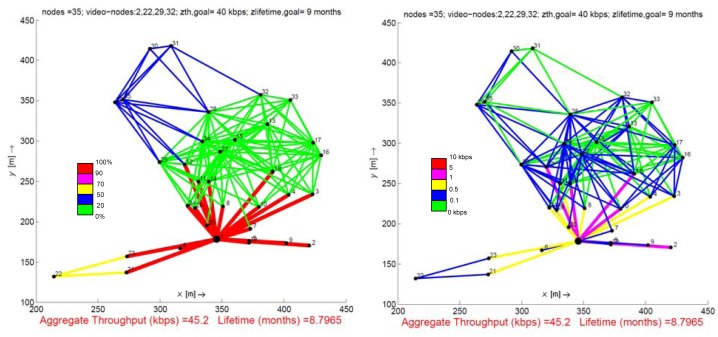
Case study that shows the GP flexibility: Although the expected network lifetime is not reached, GP obtains a result that approaches the goals without modifying the initial design parameters.

Figure 3 illustrates the results obtained for a network composed of 35 nodes where one of the goals, *z_lifetime,goal_*, is not satisfied. Solving the expression (13) with the initial goals defined by the decision maker (*z_th,goal_* = 40 kbps *z_lifetime,goal_* = 9 months), the optimization is unfeasible for this scenario, that is, the optimizer is not able to reach a solution for the goals planned. Under this premise, *most of the optimization techniques using rigid constraints (including some MO techniques such as the constraint or the linear weighted methods) stop their progress*, *returning no result at all*. A possible solution to overcome this situation is to reconsider the initial network design, modifying some of the input parameters such as the number of video sensor nodes, the traffic load they generate, the placement of the nodes or simply the value estimated for the goals. This approach implies repeating the whole process the number of times required until the results are attained, affecting negatively to the feasibility of the initial solution searched. Thanks to goal programming, this concern may be avoided, because our model switches the objective to maximize: from overachieving the goals (problem (13)) to underachieving them (Equations (14) and (15)). In particular, for the case of Figure 3, we maximize the positive deviation variable for goal *z_th,goal_* and the negative deviation for goal *z_lifetime,goal_*, that is, $\overset{N}{\underset{i=2}{\text{∑}}}\overset{N}{\underset{j=1}{\text{∑}}}d_{ij}^{+}+d_{N}^{-}$. This approach allows to adjust the lifetime solution to the underachieving level of goal *z_lifetime,goal_*. The result is a 3% deviation with respect to the lifetime goal. In this case, the decision maker confirms the design proposed what means that this solution is in accordance with the requirements of the real-problem proposed, thus validating the initial design parameters.

## 4. LOAM: Optimal Load Balancing Algorithm

Our *Optima****L*** *L****O****ad B****A****lancing Algorith****M*** (LOAM) proposal is aimed at approaching the best redistribution of the traffic load according to the connectivity results offered by the former optimization. This algorithm has to be executed by every network node with the objective of achieving the best performance for the set throughput/lifetime (as it is pointed out by the mathematical optimization) on real WMSNs. To this end, every node running LOAM distributes the data flow among all its neighbors in coverage, following the rules described in the pseudo code of Table 2 above and whose parameters are defined in Table 3. In addition, it should be noted that the energy and the number of extra messages required by LOAM (network overload) are minimum with the objective not to affect the results.

The load balancing algorithm proposed is carefully designed taking into account issues such as the low computation and reduced memory capabilities of the WMSNs nodes, and, as a result, it involves little computation overhead. In this sense, LOAM is fast and requires little memory resources to process its tasks, what is crucial to program it in real WSN devices.

The following aspects are introduced to improve the algorithm's comprehension:

1. The algorithm distinguishes between nodes on the vicinity of the sink (*l* = 1) and the remaining network nodes. These nodes only require running steps 1 to 3 to obtain a similar performance to the one resulting from the optimization results, while the rest of the nodes must execute all the algorithm's steps.
2. Any node must learn about its distance to the sink (*l*) and save in its memory the parameter *l* of the neighbors in coverage. Nodes never transmit data to other nodes with a higher value of the parameter *l* than their own value.
3. Steps 4–7 constitute a loop, as the number of iterations executing these steps increases, the algorithm converges to the numerical results obtained by the analytical optimization.
4. Step 4 indicates that nodes share energy and throughput information with their neighbors, with the purpose of selecting the best one of them (step 5). In particular, step 4 is executed for instance, when a node detects a significant decrease in its battery power level due to a large amount of data delivered during a short time interval.
5. In step 5, to make the best neighbor selection, nodes calculate the threshold *^χ^_j_*, which evaluates the residual battery level of the neighbors and the current throughput carried by them. Thus, the best solution is to select, among all the neighbors along the path to the sink, *the available neighbor with the highest battery residual level and lowest delivered throughput*.
6. The algorithm introduces the compensation factor *α*, a value between 0 and 1, intended to calculate appropriately (i) the message distribution sequence among nodes and (ii) the frequency of change of transmission path. As regards (i), the algorithm selects the neighbor node with the highest *x_j_*. In relation to (ii), when α is small, the decisive parameter is the remaining energy *E_j_* of each neighbor. In this case, the number of path switches to deliver data among neighbors is low, because the variation of energy is much slower than the variation of throughput does. On the contrary, when *α* is close to 1, the throughput is the main concern and its abrupt variations (*i.e.*, a video source starts or stops its transmission) suppose continuous path switches to transmit messages among the neighbors. For the rest of the paper, we take *α* = 0.5, which means a neutral option balancing with the same weight throughput and battery level of the neighbor nodes.
7. In the particular case that a node unexpectedly fails and therefore disappears from the network, the neighbors of the failing node are aware of this new situation when they do not receive its broadcast messages corresponding to the step 4. Under these conditions, the neighbors calculate the new thresholds, discarding the failing node. If some neighbor becomes isolated, the LOAM operation finishes for this particular node. Otherwise, the neighbors balance their loads among their neighbor nodes in coverage. In the hypothetical case that the failing node returns to be alive (orphan), it indicates this event to its neighbors by means of a broadcast message corresponding to its step 2. Finally, the LOAM algorithm running in all the nodes (neighbors and orphan) redistributes again the traffic loads fairly. However, it should be noted that failing nodes are not considered in the next simulation and test-bed scenarios in order to validate the numerical planning tool that does not take into account this algorithm behavior.

## 5. Performance Evaluation

Computer simulation helps in conducting the evaluation of the same general scenarios and requirements described in the previous sections. The results obtained here also allow to confirm the feasibility and accuracy of the numerical values achieved by the former optimization process. To this aim, we programmed our energy saving model in an ns-2 simulation environment [13] to study the energy consumed for the different topologies under concern. ns-2 is a network simulator widely used by the scientific community, which has been already verified and validated for different communication technologies, communication protocols and network topologies. For our evaluation, we start from the Zheng simulator [41]. The first Zheng simulator implementation allows the performance evaluation of a detailed environment under the IEEE 802.15.4 standard [12]. In particular, we are interested in the unslotted CSMA-CA channel access control of the IEEE 802.15.4 standard, since it facilitates the execution of multi-hop solutions on-top [42]. From this first approach, we developed the necessary ns-2 functions to assess the same scenarios as proposed in the optimization section and, therefore, to fairly compare their respective results.

To conduct our simulations, different WSN topologies consisting of a fixed number of nodes (from nine to 100 nodes) were selected from the layouts generated by the optimization study. Links are established when the distance between neighbor nodes is less than or equal to 100 m, since the maximum transmission range is set to 100 m. Furthermore, the network coordinator is the sink/base station node appointed to receive all network data. The coordinator was placed at the network labeled as node *1*. The remaining network nodes deliver information to the coordinator according to (i) the results of traffic load offered by the optimization study and (ii) the routing tables and connectivity matrixes provided by the LOAM algorithm. In both cases, the concept of duty-cycled WMSNs is maintained [43]: a node consumes energy when transmits/receives messages, operating in the ON period; the remaining time, the node keeps on the sleep mode (OFF period). MPEG-4 traffic was considered for video nodes while Constant Bit Rate (CBR) traffic was used for the sensing nodes [39] operating at the same bitrates and employing the same message sizes as the ones indicated in the numerical results subsection. In both cases nodes work at 2.4 GHz for the GSM ISM band (250 kbps nominal transmission bitrate). Finally, as most large-scale WMSN applications are deployed in outdoor environments, the free-space propagation model is a well-accepted and reasonable approach for our scenarios.

**Figure 4.**
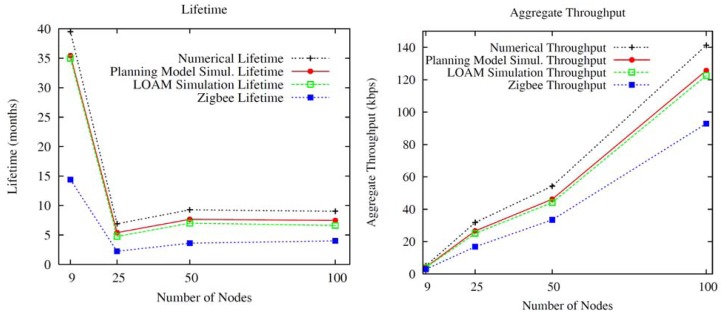
Comparison of Numerical and Simulation Results for the Lifetime and Aggregate Throughput metrics. In both figures, the simulation results show the average throughput and lifetime.

In the simulation experiments where the unique device without energy restrictions is the network coordinator, the areas managed by a few intermediate nodes (low node density in that area) and the large amount of traffic to transmit (video services) has a significant influence on both the network lifetime and the aggregate throughput. In this context, Figure 4 illustrates that networks consisting of nodes with few neighbors and supporting a high traffic load have a notable reduction of the network lifetime and throughput associated to them. The reason is twofold: (i) the increase of the number of messages that must be disseminated by the same network nodes and (ii) the increase in the number of times that the unslotted CSMA-CA mechanism is triggered. When the node density is low, a few intermediate nodes are the responsible for dispatching the messages to the sink as well as carrying out their respective monitoring tasks. Thus, other network nodes must constantly access them to transmit their information, resulting in an increase in the probability of message collision. To avoid message collisions, IEEE 802.15.4 employs the unslotted CSMA-CA for multi-hop networks. This mechanism is based on the idea that network nodes must wait for a random time in case the channel is busy. Therefore, when a node is ready for transmitting a message, it proceeds to listen to the physical medium, switching its transceiver to the ON mode. If the medium is free, the message is automatically sent but if another neighbor device is simultaneously transmitting, the node aborts its transmission attempt and triggers the unslotted CSMA-CA algorithm. This algorithm defines the new time to again attempt the transmission of the message under consideration. This process consumes energy until the node encounters the medium free and the message is delivered. As a consequence, if the number of intermediate nodes is low, the probability of sensing the channel busy increases and, therefore, more energy is invested in sending a message. Furthermore, the execution of unslotted CSMA-CA involves more time for transmitting a message, thus decreasing the aggregate throughput metric.

Figure 4 illustrates the simulation and numerical results for our planning model based on goal programming and the proposed LOAM algorithm. As can be seen, the network lifetime and aggregate throughput obtained for the LOAM algorithm almost match the values achieved by the GP optimization model. This implies that our optimal planning model based on GP may be translated to a realistic WMSN running the LOAM algorithm in every node of the network.

In addition, Figure 4 also compares the lifetime and aggregate throughput of Zigbee networks that include the conventional *Ad-hoc* On-Demand Distance Vector (AODV) [44] WSN routing algorithm. Zigbee continuously updates the routing tables of all network nodes to select the best (and unique) path between any arbitrary source and destination nodes. Network nodes belonging to a particular source-destination path must receive all the data messages of its respective predecessor node and retransmit them to the subsequent one. This procedure causes that nodes participating in an active path consume more energy than the remaining nodes, what leads to network nodes with a substantial energy disparity. This fact has a very negative impact on the network lifetime. An appropriate redistribution of traffic flows by, for instance, selecting different paths for a same source-destination pair as our algorithm does, is clearly advantageous to save energy. Thereby, the lifetime obtained by Zigbee is considerably less than that offered by our energy saving model. Regarding the throughput, the distribution of the traffic flows according to our algorithm involves different paths between source and destination, thus reducing the number of occasions that the same intermediate nodes take part in transmitting the information, which also results in a lower probability of collision. However, in static Zigbee networks, the paths source-sink usually include the same intermediate nodes. It means that the remaining nodes transmit data through the same intermediate nodes causing a higher number of collisions and, therefore, using the unslotted CSMA-CA algorithm more times. This fact reduces the time dedicated to dispatch messages and, as a result, the aggregate throughput.

Figure 4 also reveals that the optimization study represents an upper bound. As it is shown, for a same topology, the lifetime and the aggregate throughput obtained by simulation is slightly less than the one accomplished by the optimization analysis. This behavior is mainly because the simulation includes in its evaluation the effect of the IEEE 802.15.4 MAC and PHY layers. Thereby, for a same scenario, nodes consume more energy and achieve less throughput in the simulation, since the GP optimization procedure obviates real processes, such as the channel access control or the retransmission of messages. However, the simulation results are very close to the numerical ones, validating, therefore, the optimization study. Figure 4 shows that the lifetime plots for the planning model simulation and LOAM algorithm are very close, almost coincident. This fact indicates a little network overload associated to all the LOAM broadcast messages in comparison with the video and data traffic transmission.

## 6. Experimental Results: Crop Network Deployment

This part describes implementation issues taking into consideration the multi-objective solution stated in the previous sections. It is aimed at reproducing the same scenario composed of nine nodes that was generated in the design section for the optimization study. Therefore, the network topology, traffic type, generation rates, frame size and all the features previously introduced can be tested on real devices. The objective is twofold: (i) to validate the numerical results and also the simulation environment of the IEEE 802.15.4 standard employed for the deployments presented in this work and (ii) to build a test-bed/trial scenario in order to evaluate the throughput and the network lifetime metrics experimentally, and to gain insight into the network real feasibility.

Under these considerations, different hardware and software components have been employed and/or developed, providing the abilities for crop sensing monitoring of physical parameters [45] and video transmission with the appropriate sensors. As regards hardware, we used three different devices: two commercial ones (MicaZ and Imote2 produced by MEMSIC) and one prototype developed by us (denoted as BS-101), all of them presented in work [46]. In particular, as sensing node we employ the MicaZ [16] device and the MTS300 sensor board, which integrate in a same hardware platform the radio module, micro, memory and sensors as temperature and humidity (used in our deployment). The Imote2 mainboard, including a battery board, and the Imote2 Multimedia Board IMB400 [14] constitute a commercial wireless sensor network device specifically designed to develop applications that need reliable wireless connections and high CPU requirements (for instance, video applications). Finally, the base station BS-101 receives the data information from the remaining network nodes and dispatches it to distances up to 7 km, where decision makers analyze the results received. All devices support IEEE 802.15.4 communications (radio module of Texas Instruments CC2420 at 2.4 GHz and 250 kbps nominal transmission bitrate), what facilitates its compatibility and network integration.

Software components, in turn, must be compatible with the hardware, and they also have to enable the development of sensor applications and the LOAM algorithm described in section 4. In particular, these implementations have been developed using TinyOS (version 2.1.1) [47] and the nesC language [48]. TinyOS is one of the most widely employed operating system for WSN. The software developed has been uploaded to the website http://labit301.upct.es/∼ajgarcia/ along with the user's manuals. The goal is that any developer may reproduce our project using the hardware previously exposed and the provided software.

**Figure 5.**
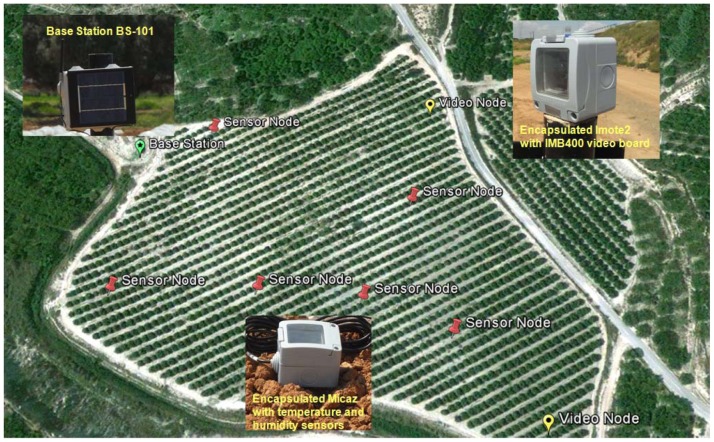
Test-bed/Trial scenario in Campo de Cartagena (Spain). Real devices are exactly deployed as indicated by the design study

Finally, in order to validate the throughput and network lifetime achieved by simulation and analysis (Figure 4) as well as the proper operation of the devices, a WMSN was deployed for a field trial (Figure 5) following the layout depicted in Figure 1-a. Nodes were deployed in an agricultural farming environment to monitor a broccoli crop located in Campo de Cartagena. Six MicaZ devices including temperature and humidity sensors and two Imote2 nodes incorporating the video sensor were installed in this crop, alongside with a base station node covering an area of 20.000 m^2^.

The sensors installed are responsible for: (i) video-surveillance to supervise the state of the plants as well as their fruits and (ii) learning about the seed germination, the growth of the plant, the leaf size, the flowering period, *etc.*

The location of each node follows the scenario illustrated in Figure 1-a. The final placement of video sensors is aimed at obtaining the major visual camera coverage and good radio-signal coverage of the crop. The location selected for the base station avoids the signal loss caused by the plants or any other type of trees in the path to the end-user. In addition, we also employ the results obtained for the LOAM simulation under the same topology conditions (9 nodes) to generate a scheduled duty-cycle in each node. These results allow to reproduce the exact number of messages that the nodes must send or receive in a time interval. To execute these tasks, nodes remain in the ON period until the last message is sent/received appropriately. When this operation is completed and before nodes switch to OFF (sleep mode), the exact time instant of the following ON period is updated in order to know when the nodes must wake up again.

**Figure 6.**
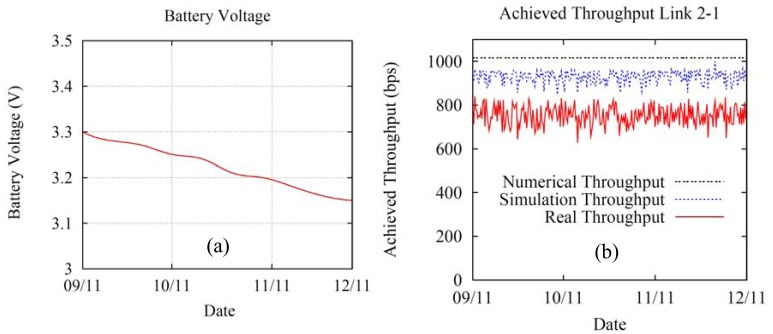
Metrics for the MicaZ labeled as number 2 (**a**) Battery voltage consumption during an agronomic cycle (**b**) Average throughput achieved every 8 hours for link 2-1. The Simulation and Real cases are programmed to use the proposed LOAM algorithm.

Figure 6 shows the throughput and lifetime for the network node labeled as 2, a MicaZ device. It is selected since its throughput and power consumption is the greatest of the network, as it is calculated by the numerical solution. Under these conditions, node 2 carries a larger amount of the network traffic load (in comparison with the remaining nodes) and, as a result, its battery depletes sooner. To conduct our test, nodes 2 and 1 were reprogrammed to run a piece of software that measures and stores the energy consumption of node 2, and calculates the average achieved throughput every 8 hours for the link 2-1. Figure 6(a) plots the energy consumed by node 2 from September 2011 to November 2011 in the crop. After three months of operation (one complete agronomic cycle for the broccoli crop) and for the design parameters introduced in the numerical results subsection, the batteries' voltage drops to 3.15 V, which is a decrease of 0.15 V from 3.3 V. This drop does not affect the normal operation of the MicaZ since it provides a working range of 2.7–3.6 V [49]. Therefore, it can be stated that the system works appropriately during the entire crop cycle. Figure 6(b) represents the average achieved throughput per day for link 2-1. The results are satisfactory because they reasonably match those obtained by the simulations and the analytical study under the same design parameters (number of video/sensing nodes, message size, traffic generated by node, placement of the nodes, *etc.*). Finally, we have also measured the computation overhead introduced by the LOAM process at the nodes, which can be considered negligible. This is due to the low number of LOAM operations performed in the network nodes. For instance, in our test-bed and for each node, we run LOAM one time each 20 s, what means battery consumption for these operations around 1% with respect to the total consumption. If LOAM is operating one time each 60 or 120 s, the computation overhead effect is even much lower.

## 7. Conclusions

In this paper, we present a mathematical model which optimizes two conflicting metrics for the WMSN: throughput and network lifetime. Goal Programming based on Multi-objective optimization is used to achieve this purpose, simultaneously maximizing both performance figures. This, to the best of the authors' knowledge, has never been considered in previously published research. Unlike other multi-objective optimization techniques applied in WSNs, when the optimal solution is not reachable, our model may obtain an appropriate outcome without modifying the initial values for the design parameters. Thus, we offer a powerful planning solution valid for any static arbitrary topology (cluster, grid, random, *etc.*) that fairly balances the traffic load of each communication path. As a consequence, the data gathered at the base station is maximized, what offers to the end-users the best performance for both video and sensing data. Additionally, an *Optima****L*** *L****O****ad B****A****lancing Algorith****M*** named LOAM running in each network node offers similar results for the set lifetime/throughput to those returned by the planning solution.

To validate the numerical solution and the correct behavior of the LOAM algorithm, a simulation framework and test-bed have been developed. The simulator allows us to reproduce the same scenarios as the ones proposed in the numerical design subsection. The simulation framework includes the planning model and the LOAM *algorithm* as well as the entire IEEE 802.15.4 protocol stack. Test-bed presents a real scenario formed by video, sensing and base-station nodes that were deployed in a broccoli crop to measure the energy consumed and the achieved throughput of the most critical network node. In the test-bed, each node implements and runs the corresponding software modules according to its service/application as well as the LOAM algorithm. Effects such as the connectivity between nodes or the reliability of the links are now included. Simulation and experimental results show that the metrics measured suffer a slight reduction in comparison with the results obtained by the numerical approach.

Our future research in this field is aimed at further improving our mathematical model, providing a better approach to real implementations. The inclusion of new constraints and/or objectives related with the IEEE 802.15.4 medium access mechanism or with the routing issues can be addressed in this direction, but the mathematical complexity/computing time associated as well as the convergence to a feasible solution are relevant issues to take into consideration. On the other hand, information about any intermediate node that depletes its battery must be known by the remaining of the network nodes by means of the execution of *LOAM algorithm* which also recalculates, at run time, the new traffic load per link. This calculation, made at each node (which may be placed away from the “dead” node), takes into consideration the loss of those links with “dead” nodes. This process will allow to *LOAM* to find out the path to the destination with the best performance. Thus, the network can operate until a source node is isolated therefore extending the network lifetime. As a crucial requirement, this process must not incur in an excessive waste of energy, memory and processing capacity.

## Figures and Tables

**Table 1.** Description of each parameter for the MO model under study.

**Parameter**	**Description**
*th_ij_*	Achieved throughput of the link *ij* (b/s)
${\text{th}}_{ij}^{V}$	Achieved throughput due to video sources in the link *ij* (b/s)
${\text{th}}_{ij}^{D}$	Achieved throughput due to sensing nodes in the link *ij* (b/s)
*N*	Number of nodes of the WMSN
Λ	Aggregate throughput (b/s)
*K_ij_*	Parameter that indicates the existence of a link between nodes *i* and *j*. If *K_ij_* = 1, there is a link; otherwise *K_ij_* = 0
*S_i_*_→_*_j_*	When a message is transmitted to a node, parameter that indicates if this node is nearer to the sink than the transmitter one. If *S_i_*_→_*_j_* = 1, the node selected is nearer to the sink; otherwise *S_i_*_→_*_j_* = 0
*_ν_*	Node transmission bit rate (b/s)
_Ψ_	Maximum WSN/WMSN transmission bitrate (b/s)
*T_NETWORK_*	Network Lifetime
*E_tx_*	Energy consumed in the transmission of a message in joules per bit, including the energy wasted by the processor module, transceiver module and the transition states between the sleep state to the transmission mode
*E_rx_*	Energy consumption to receive a message in joules per bit, including the energy wasted by the processor module, transceiver module and the transition states between the sleep state to the reception mode
*E*_Re*sidual*_	Energy consumed by a node in the time periods of no message transmission/reception, that is during the sleep mode. It is expressed in joules per second
*E_source_*	Initial energy for any arbitrary node in joules

**Table 2.** Algorithm 1: LOAM pseudo code.

**Algorithm 1.**
Network establishment Stage: 1. Index *l*=*0* is assigned to the sink node, which transmits a broadcast message with this value *l*=*0*, 2. Do in all the network nodes Any arbitrary node *i* with index 1≤*l*≤*d* receives the broadcast messages with value *l-1*, then it transmits a broadcast message with index *l*, ∀*l* ∈ [1,*d*], ∀*i* ∈ *N* Any arbitrary node *i* creates a vector *H_i_*, which is fulfilled with the respective values *l* received from its neighbor nodes *j* in case *th_ij_* > 0 or otherwise zero, ∀*l* ∈ [1,*d*], ∀(*i*,*j*) ∈ *N* Transmission Node Selection Stage for all network nodes: 3. Given an arbitrary node *i*, **if** *l* =*1*, this means that node *i* is a neighbor node of the sink, then it can transmit directly to the sink *l*=*0*. **End algorithm**. 4. ***else***, 4.1. Node *i* with assigned index *l* broadcasts a message with its current outgoing throughput $\sum_{j=1}^{N}{\text{th}}_{ij}$ and *E_i_* ∀*l* ∈ [1,*d*], ∀(*i*,*j*) ∈ *N* 4.2. Node *i* stores in its memory all the received values $\sum_{k=1}^{N}{\text{th}}_{jk}$ and *E_j_* ∀*j* ≠ *i*, ∀*k* ≠ *j*, ∀*l* ∈ [1,*d*], ∀(*i*,*j*,*k*) ∈ *N* 5. ***for*** *j*=*1 to N* Node *i* calculates the thresholds (*χ_j_*) for all its neighbor nodes *j*|*H_i_*(*j*) > 0, as follows: $\begin{array}{ccc} \chi_{j}=\alpha \cdot \left(\psi -\overset{N}{\underset{k=1}{\text{∑}}}{\text{th}}_{jk}\right)+\left(1-\alpha \right)\cdot E_{j} & \forall j\neq i,\forall k\neq j, & \alpha \in \left[0,1\right],\forall (i,j,k)\in N \end{array}$ 6. Node *i* selects node *j* to transmit data messages | max(*χ_j_*), ∀(*i*,*j*) ∈ *N* 7. Go back to step 4.

**Table 3.** Parameters defining the LOAM algorithm.

**Parameter**	**Description**
*th_ij_*	Achieved throughput for the link *ij* (bits/s) where *j* is a neighbor node of *i*
*th_jk_*	Achieved throughput for the link *jk* (bits/s) where *k* is a neighbor node of *j*
*l*	Index denoting the distance (number of hops) from an arbitrary node to the sink node (*l* = 0 is assigned to the sink). A similar index is also used in other works such as [40]
d	Maximum distance to sink node, also called network dimension.
H(j)	Vector describing the distance of neighbor nodes *j* (of any node *i*) to the sink
*_E_i_, E_j*	Parameter that indicates the remaining (residual) energy of nodes *i* or *j*, respectively
*χ_j_*	Threshold to make a decision about which is the best neighbor of any node *i* to forward data
*α*	Compensation factor between throughput and energy consumption
